# Movement of the external ear in human embryo

**DOI:** 10.1186/1746-160X-8-2

**Published:** 2012-02-01

**Authors:** Miho Kagurasho, Shigehito Yamada, Chigako Uwabe, Katsumi Kose, Tetsuya Takakuwa

**Affiliations:** 1 Human Health Science, Graduate School of Medicine, Kyoto University, 606-8507, Sakyo-ku Shogoin Kawahara-cyo 53, Kyoto, Japan; 2 Congenital Anomaly Research Center, Kyoto University Graduate School of Medicine, Kyoto, 606-8507, Japan; 3 Institute of Applied Physics, University of Tsukuba, Ibaragi, 305-8573, Japan

**Keywords:** External ear, Internal ear, Three-dimensional kinetics, Human embryo, MR imaging

## Abstract

**Introduction:**

External ears, one of the major face components, show an interesting movement during craniofacial morphogenesis in human embryo. The present study was performed to see if movement of the external ears in a human embryo could be explained by differential growth.

**Methods:**

In all, 171 samples between Carnegie stage (CS) 17 and CS 23 were selected from MR image datasets of human embryos obtained from the Kyoto Collection of Human Embryos. The three-dimensional absolute position of 13 representative anatomical landmarks, including external and internal ears, from MRI data was traced to evaluate the movement between the different stages with identical magnification. Two different sets of reference axes were selected for evaluation and comparison of the movements.

**Results:**

When the pituitary gland and the first cervical vertebra were selected as a reference axis, the 13 anatomical landmarks of the face spread out within the same region as the embryo enlarged and changed shape. The external ear did move mainly laterally, but not cranially. The distance between the external and internal ear stayed approximately constant. Three-dimensionally, the external ear located in the caudal ventral parts of the internal ear in CS 17, moved mainly laterally until CS 23. When surface landmarks eyes and mouth were selected as a reference axis, external ears moved from the caudal lateral ventral region to the position between eyes and mouth during development.

**Conclusion:**

The results indicate that movement of all anatomical landmarks, including external and internal ears, can be explained by differential growth. Also, when the external ear is recognized as one of the facial landmarks and having a relative position to other landmarks such as the eyes and mouth, the external ears seem to move cranially.

## Introduction

External ears, one of the major face components, show an interesting movement during craniofacial morphogenesis in human embryo. The external ear is evidently recognizable after Carnegie stage (CS) 16, and its movement has been described in most embryology textbooks as well [1-5]. The external ears are contained in the lower neck region at CS 17. With the development of the face structure, they ascend to the side of the head at the level of the eyes [6-10]. Streeter [11] has described the essential and precise external movement. The two auricular areas nearly meet in the mid-ventral region in a 6 mm-embryo; they are gradually moved laterally and dorsally. Streeter suggested that the movement of the external ear might be relative rather than real because the external ear is located at the side of the mouth during the development. In the recent study, Gasser [12] proposed that positional changes of the developing structures could be explained by differential growth (i.e. changes in the size and shape of the embryo and its parts) rather than migration (i.e. structures moving from one region of the embryo to another). Gasser demonstrated the evidence by showing the following three examples: sclerotome formation from the somite, spinal ganglion formation from the neural crest, and thymus, thyroid and parathyroid gland formations from pharyngeal endoderm. He emphasized the use of more centralized and less mobile reference points and comparison of both external and internal structures together in the identical magnification for better understanding of the positional changes of the developing structures.

The present study was aimed at resolving the question - could such a dynamic movement as external ear also be explained by differential growth? MR imaging data of human embryos from the Kyoto Collection of Human Embryos [13]; http://bird.cac.med.kyoto-u.ac.jp] was used for tracing the 3D absolute position of the anatomical landmarks. The MR data had the advantage of comparing facial structures between different stages with identical magnification. Data revealed that positional changes of external and internal ears that occur during morphogenesis could be explained by differential growth.

## Materials and methods

### Human embryo specimens

Around 44,000 human embryos (constituting the Kyoto Collection) were historical specimens collected and stored at the Congenital Anomaly Research Center of Kyoto University [14-16]. In most cases, pregnancy was terminated during the first trimester for socioeconomic reasons under the Maternity Protection Law of Japan. Some of the specimens (~20%) were undamaged, well-preserved embryos. When the aborted materials were brought to the laboratory, the embryos were measured, examined, and staged using the criteria of O'Rahilly and Müller [10]. Approximately 1,200 well-preserved human embryos diagnosed as externally normal at CS 13 to CS 23 were selected for MR microscopic imaging. The conditions used to acquire the MR images of the embryos are described elsewhere [17,18].

### MR image processing and selection of datasets

3D MR image datasets for each embryo were initially obtained from 256 × 256 × 512 voxels. Each dataset was first converted into a two-dimensional (2D) stack and saved as an audio video interleave (.avi) file format using software ImageJ™ (version 1.42q, National Institutes of Health, Bethesda, MD). Sequential 2D images were resectioned digitally and 3D images were reconstructed using the software OsiriX™ (version 3.7.1, Pixmeo SARL, Geneva, Switzerland). Both 2D and 3D images were carefully observed and selected according to the following conditions: 1) no obvious damage or significant anomaly present in the external appearance; 2) body axes maintained in the original form, i.e. not deformed artificially during fixation and preservation; 3) sufficiently high quality of reconstructed 2D images to properly extract the organs and tissues. For the present study, 171 samples between CS 17 and 23 were selected from all 1,200 MR image datasets based on the criteria described above. The number of cases for each CS was distributed between 18 and 30.

### Anatomical landmarks

**Figure 1:**
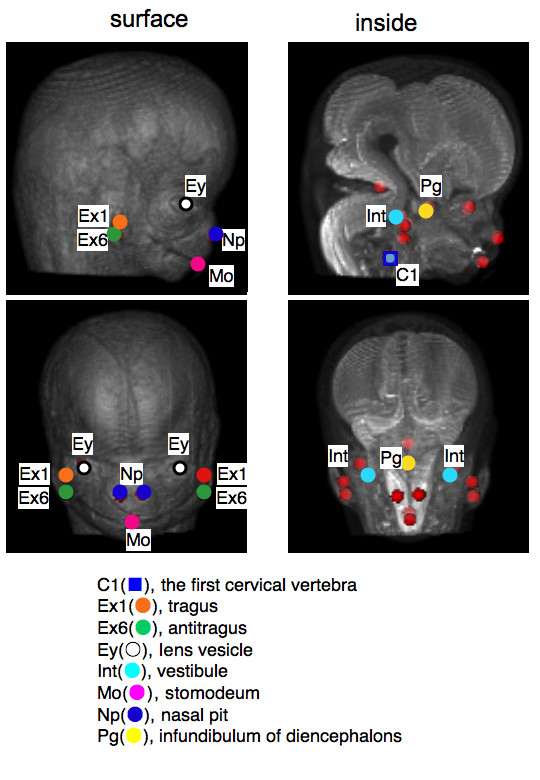
**The 13 landmarks selected for present study**. Landmarks were expressed on volume rendering 3D images of the head region for surface and maximum intensity projection for inside constructed with software OsiriX.

The 3D coordinate was initially given for 13 selected landmarks by examining the position of the voxel on 2D sequential and 3D images using OsiriX (Figure 1). The selected 13 landmarks were as follows: bilateral auricular hillock on the first cranial arch which becomes tragus later (Ex1), bilateral auricular hillock on the second cranial arch which becomes antitragus later (Ex6) and vestibule (Int) as representative external and internal ear landmarks; stomodeum which becomes a part of mouth (Mo), bilateral nasal pits (Np), bilateral lens vesicles which become a part of eyes (Ey) as external anatomical landmarks, and infundibulum of diencephalons (later pituitary gland) (Pg) and cranial region of the first cervical vertebra (C1) as internal anatomical landmarks.

#### Evaluation of the position of anatomical landmarks

**Figure 2:**
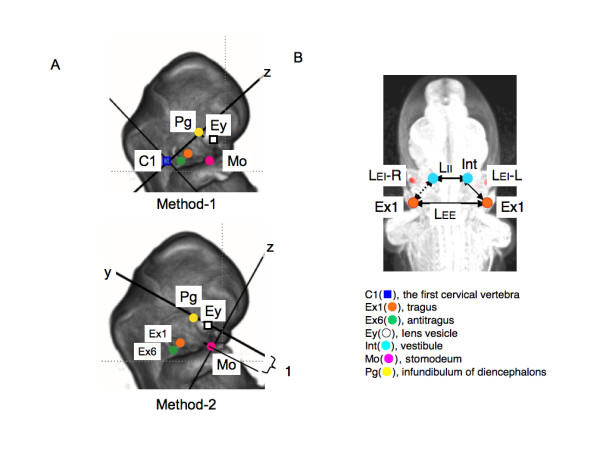
**Lateral view showing reference axes, anatomical landmarks and morphometry in present study (A) Reference axes used in Method-1 and Method-2**. Reference axes and landmarks were expressed on volume rendering 3D images of the head region constructed with software OsiriX. (B) Anatomical landmarks and morphometry measured in present study. Landmarks were expressed on maximum intensity projection 3D images of the head region constructed with software OsiriX for inside viewing. Distances between bilateral Ex1s, bilateral Ints and collateral Ex1 and Int were defined as L_EE_, L_EI_, and L_EI_, respectively.

Two kinds of methods were used to evaluate and analyze the position of the anatomical landmarks in the present study (Figure 2A). Method-1 was used for evaluating the absolute position while Method-2 was for the relative position of each landmark.

##### Method-1 (Measurement of the absolute position)

The 3D absolute position of the anatomical landmarks from MR image data was used to compare the position of each anatomical landmark between the different stages with identical magnification. The line connecting C1 and Pg was defined as reference axis (Z-axis) of 3D orthogonal coordinate for this purpose. Both C1 and Pg are less mobile internal structures close to notocord and detectable during the development [1,19]. Distances between bilateral Ex1s, and collateral Ex1 and Int were calculated and defined as L_EE _and L_EI _respectively (Figure 2B). The middle point of the collateral Ex1 and Ex6 were defined as Exm.

##### Method-2 (Measurement of the relative position)

The relative position of the external ears with other landmarks during craniofacial morphogenesis was observed from the frontal position of the face at each stage. For this purpose, the line connecting the middle point of the bilateral Eys and Pg was defined as reference axis (X-axis). The vertical line to the X-axis which contained Mo was defined as Z-axis of 3D orthogonal coordinate. The XY plane defined by this method was almost parallel to the structures at the base of the skull, which divide the area between the neurocranium and viscerocranium [1]. Further, the distance between the middle point of bilateral Eys and Mo was kept constant at one so as to adjust the expansive growth of the face (Figure 2A).

This study was approved by The Committee of Medical Ethics of Kyoto University Graduate School of Medicine, Kyoto, Japan (E986).

## Results

### Absolute position of internal and external ear and anatomical landmarks

**Figure 3:**
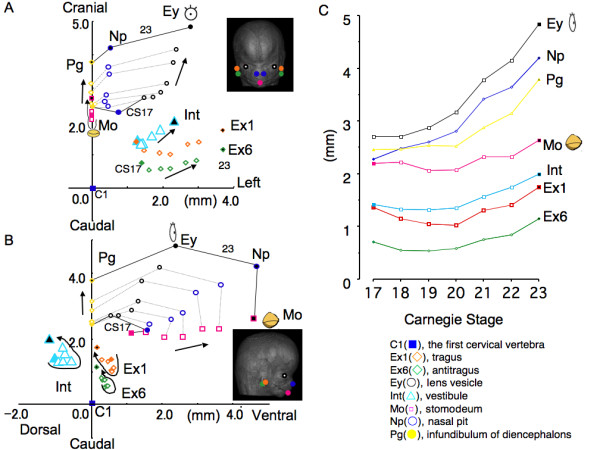
**Movement of external and internal ear and anatomical landmarks during development in the orthogonal coordinate defined in Method-1**. Frontal view (A) and lateral view (B). The line which connects the pituitary gland and the first cervical vertebra was reference axis (Z-axis) (See also Figure 2). (C). Movement of anatomical landmarks along the reference (Z)-axis.

The 3D absolute position of the anatomical landmarks was shown using Method-1. All landmarks moved away from the origin (C1) by frontal view (Figure 3A). The line connecting Pg, Ey and Np became longer, which might indicate swelling of the face. Ex1, Ex6 and Int also moved away from the origin (C1) like other landmarks, except that the movement of Int between CS 17 and CS 20 was slow.

By right lateral view, the lines connecting Pg, Ey, Np and Mo, resembling the side view of the swelling face, also became larger as development proceeded (Figure 3B). The movement of Ex1, Ex6 and Int was different from that of the other landmarks. They rotated clockwise for Ex1 and anti-clockwise for Ex6 and Int around CS 17 and CS 21; after that, they moved dorso-cranially.

The position of each landmark in the cranial direction between CS 17 and CS 23 was demonstrated (Figure 3C). All landmarks changed positions smoothly and gradually during development. As for Int, Ex1, and Ex6, the movement of the position along the cranial/caudal direction was very limited.

### Distance and 3D relationship between internal and external ear

**Figure 4:**
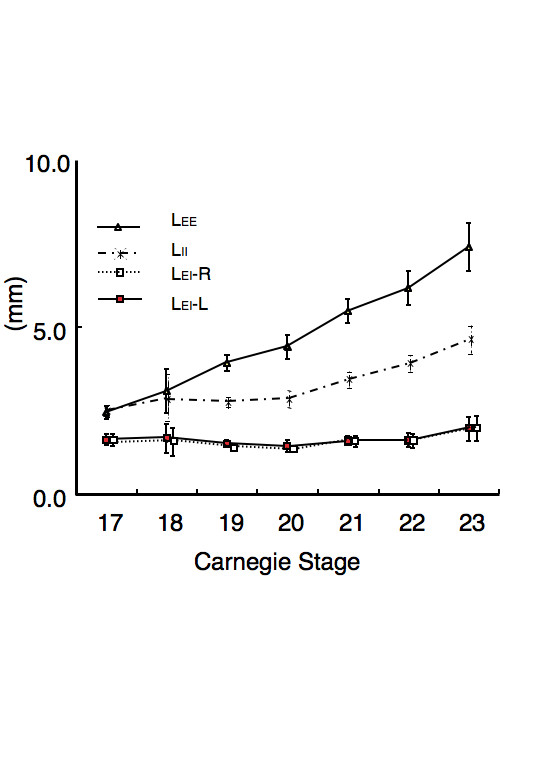
**Distance between external and internal ears**. Distances between bilateral external ears (L_EE_), bilateral internal ears (L_II_), and left internal and external ears (L_EI_-L) and right internal and external ears (L_EI _-R) are shown according to CS. (See also Figure 2C.) L_EE _and L_II _increase with development, whereas, L_EI_-L and L_EI _-R remain approximately constant between CS 17 and CS 23

L_EE _and L_II _increased as CS proceeded (Figure 4). L_EE _at CS 17 was 2.43+/-0.20 mm (mean +/-SD), and reached 7.38+/-0.72 mm at CS23. L_II _at CS 17 was 2.52+/-0.18 mm, and reached 4.63+/-0.41 mm at CS 23. L_EE _may correspond to the lateral growth of the face, whereas L_II _corresponds to the lateral thickness of the neural tube. Interestingly, both L_EI_-L and L_EI_-R were kept approximately constant, with distribution between 1.42 and 2.01 mm.

**Figure 5:**
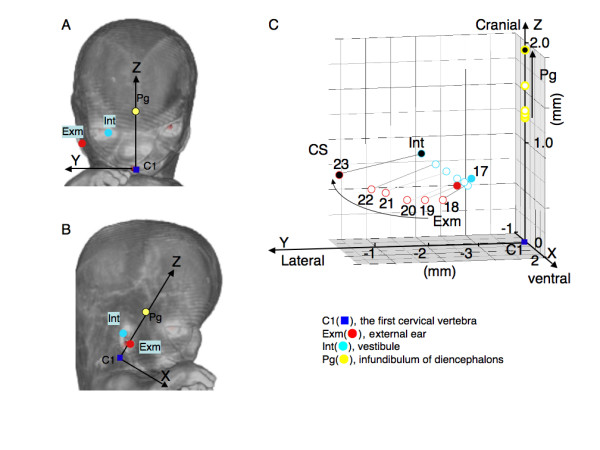
**Relationship between right external and internal ears during development**. Axes and anatomical landmarks are shown on frontal (A) and lateral (B) views of volume rendering images. (C) Three dimensional graph showing the relationship between right external and internal ears during development.

Three-dimensionally, the external ear (Exm) located vent-caudal parts of the internal ear in CS 17, and then moved mainly laterally (Figure 5). Exm rotated approximately 49 degrees during CS 17 and CS 23 when Int was the origin (data not shown).

### Relative movement of external and internal ear

**Figure 6:**
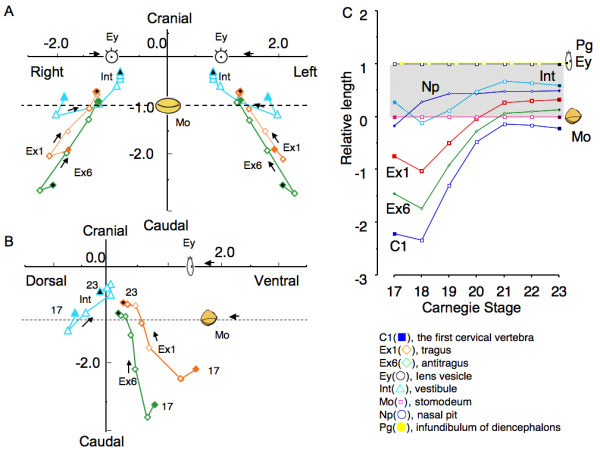
**Movement of external and internal ear and anatomical landmarks during development in orthogonal coordinate defined in Method-2**. Frontal (A) and left lateral (B) aspects of the face. Line connecting the middle point of the bilateral lens vesicle (Eys) and pituitary gland (Pg) was defined as reference axis (X-axis). The vertical line to the X-axis, which contained Stomodium (Mo) was defined as Z-axis of 3D orthogonal coordinate. The relative position of anatomical landmarks including Ex1, Ex6 and Int is compared with the distance between XY plane and Mo at each CS. (C) Movement of anatomical landmarks along the cranial/caudal (Z)-axis in the orthogonal coordinate in Method-2

The relative movement of anatomical landmarks including Ex1, Ex6, and Int was demonstrated using Method-2 (Figure 6). Most landmarks gathered toward the origin as CS proceeded. The relative movement was noticeably larger in Ex1, Ex6, and Int than in Eys, Np, and Pg. Ex1 and Ex6 moved from the caudal lateral ventral region toward the origin. They moved with high speed between CS 17 and 20, and then located and almost stayed between Eys and Mo after CS 21 (Figure 6C). Int migrated from the caudal dorsal lateral region and almost stayed there after CS 21 as well.

## Discussion

In a recent study, Gasser [12] proposed that some events of mammalian embryogenesis can result from differential growth. Gasser pointed out that dramatic changes occur in size and shape of the embryo and its internal structures but these changes were not considered in past studies that described migratory movements. The present study was performed to reveal whether the movement of human external ears could also be explained as differential growth.

As a principle of physics, all movements occur in relation to a reference point. Thus, it is important to select appropriate references. Gasser [12] claimed that the ideal reference point would be one in the center of the mass, i.e. a centroid. Even though no such consistently occurring point exists in developing embryos, any point that is more centrally located would move less in relation to surrounding structures and therefore would be more suitable. Two methods were used to evaluate the position of the anatomical landmarks in the present study. Method-1 was planned according to Gasser's opinion. The authors selected both C1 and Pg as an ideal reference axis. These were the most central of the embryonic structures located along the notochord. The notochord is an important structure for vertebrate animals, determining the cranial/caudal axis and dividing left and right. It runs along the neural tube and the anterior tip of the notochord reaches an area where the Pg starts to develop [1,19]. C1 body segment is used as one of the reference points in Gasser's study [12]. Another reason for selecting these points was that both were clearly detectable in all stages analyzed.

Movement of 13 anatomical landmarks displayed on the orthogonal coordinate in Method-1 demonstrated that they spread out within the same region as the embryo enlarges and changes shape. The result indicated that the positional changes that occur during the movement of the all anatomical landmarks including external and internal ears could be explained by differential growth.

The external ear did move mainly laterally, but not cranially. In the previous study, Streeter [11] had described that external ears gradually moved laterally and dorsally. As for cranial movement, Streeter suggested that the movement might be relative rather than real because the external ear is located at the side of the mouth during the development. Movement of the external ears along the dorsal/ventral axis was different from that of other anatomic landmarks such as the eyes, nose and mouth. The difference may result from the prominences they are derived from. Face components are formed from five facial primordia which appear early in the fourth week around the Mo [6-9,20,21]. Ey and Np were derived from the nasal prominence located cranial region of the Mo, while Ex1 and Ex6 were from the first and second pharyngeal arches respectively, which were located caudal region of the Mo.

In the present study the movement of the internal ear was limited in all directions. The internal ear develops from the otic placode that appears on either side of the neural tube at the level of the future hindbrain or metencephalon [1-4]. Sensory nucleoli of the vestibulocochlear complex are located close to the inner ear. Therefore, it seems hard to move the internal ear from the initial place. The distance between the external and internal ear was almost constant. This was anticipated, considering that all components of the ear relating to the sound-conducting apparatus of the middle and external ears and of the neurosensory structures of the internal ear develop simultaneously throughout development [5].

Surface reference point, i.e. Eys and Mo was adopted in Method-2 for comparison. To observe from the frontal position of the face in all stages, internal structure Pg was also selected. As expected, the movement in the orthogonal coordinate resembled our institutional macro-observation. The present data demonstrated the evident movement of the external ear from the ventral lateral caudal region to the region between the eyes and mouth.

**Figure 7:**
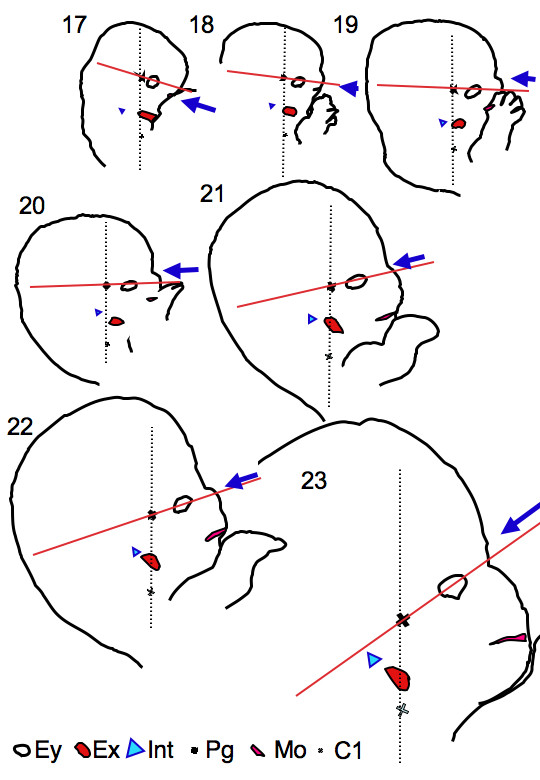
**Lateral view of embryos between CS 17 and CS 23 showing craniofacial morphogenesis**. The dashed line indicates the reference axis (Z-axis) in Method-1 and the red line indicates the reference axis (X-axis) in Method-2. The arrow indicates the frontal side of the face defined in Method-2. The following anatomical landmarks are drawn: external ear (Ex), internal ear (int), eye (Ey), mouth (Mo), pituitary gland (Pg), and first cervical vertebra (C1). Magnification is identical in all stages. The frontal position defined by Method-2 rotated as stages proceed. Note that both external and internal ears were located at similar positions between Pg and C1 between CS 17 and 23

Very different conclusions may be reached about movements of structures by selecting different references in the two methods. The difference may be owing to the following reasons:

1) The magnification of the embryo in all stages was identical in Method-1 while it was adjusted in Method-2. As a consequence, the relative position to the representative landmarks, Eys and Mo may be emphasized in Method-2. Undoubtedly, Eys and Mo are the most important landmarks to recognize the face and these are the landmarks by which the relative position of other landmarks was recognized.

2) There was a gross change of angle between the reference axes used between CS 17 and CS 23 (Figure 7). The change of angle may result from the formation of the mandibular apparatus and the structures at the base of the skull as described in Streeter's study [11]. Pharyngeal arches, especially the first arch, play an important role in the formation of the face [1-5]. In addition to the external ear, many landmarks such as the maxilla, mandible, and a part of middle ear (the incus and malleus) are formed. The development of the mandibular apparatus may move the center of the face in the caudal direction, as well as push the external ear to the lateral side, as both are derived from first pharyngeal arches. Abnormal development of the components of the first pharyngeal arch results in various congenital anomalies of the face including mandible and ears [22]. The development of the structures at the base of the skull and its contents, i.e. the brain, is another important factor resulting in the change of the angle. Chromosomal abnormalities, particularly trisomy 13 and trisomy 18 [22-25], are some congenital anomalies which show low-set ears. These syndromes express various symptoms including mental deficiency and small head and jaw. The abnormal development of the neurocranial structures in these cases may also affect the base of the skull, resulting in the symptoms of low-set ears as well.

## Conclusions

The results indicate that movement of all anatomical landmarks, including external and internal ears, can be explained by differential growth. Also, when the external ear is recognized as one of the facial landmarks and having a relative position to other landmarks such as the eyes and mouth, the external ears seem to move cranially.

## Abbreviations

(3D): Three-dimensional; (2D): Two-dimensional; (CS): Carnegie Stages; (Ex1): Tragus; (Ex6): Antitragus; (Int): Vestibule; (Mo): Stomodeum; (Np): Nasal pit; (Ey): Lens vesicle; (Pg): Infundibulum of diencephalons; (C1): Cranial region of the first cervical vertebra; (L_EE_): Distance between bilateral Ex1s; (L_EI_): Distance between collateral Ex1 and Int; (Exm): The middle point of antitragus and tragus

## Competing interests

The authors declare that they have no competing interests.

## Authors' contributions

MK has made substantial contributions to conception and design, acquisition of data, and statistic analysis. SY have been involved in drafting the manuscript or revising it critically for important intellectual content. CU has made substantial contributions to acquisition of data and maintenance of the human embryos specimen. KK have made substantial contributions to acquisition of data especially MR imaging. TT has made substantial contributions to conception and design and general supervision of the research group. All authors read and approved the final manuscript.
